# Balance control and anti‐gravity muscle activity during the experience of fear at heights

**DOI:** 10.1002/phy2.232

**Published:** 2014-02-18

**Authors:** Max Wuehr, Guenter Kugler, Roman Schniepp, Maria Eckl, Cauchy Pradhan, Klaus Jahn, Doreen Huppert, Thomas Brandt

**Affiliations:** 1 German Center for Vertigo and Balance Disorders (DSGZ), University of Munich, Munich, Germany; 2 Institute for Clinical Neurosciences, University of Munich, Munich, Germany; 3 Department of Neurology, University of Munich, Munich, Germany

**Keywords:** Balance control, fear of heights, muscle activity, muscle co‐contraction, visual height intolerance

## Abstract

Fear of heights occurs when a visual stimulus causes the apprehension of losing balance and falling. A moderate form of visual height intolerance (vHI) affects about one third of the general population and has relevant consequences for the quality of life. A quantitative evaluation of balance mechanisms in persons susceptible to vHI during height exposure is missing. VHI‐related changes in postural control were assessed by center‐of‐pressure displacements and electromyographic recordings of selected leg, arm, and neck muscles in 16 subjects with vHI while standing at heights on an emergency balcony versus standing in the laboratory at ground level. Characteristics of open‐ and closed‐loop postural control were analyzed. Body sway and muscle activity parameters were correlated with the subjective estimates of fear at heights. During height exposure, (1) open‐loop control was disturbed by a higher diffusion activity (*P* < 0.001) and (2) the sensory feedback threshold for closed‐loop control was lowered (*P* < 0.010). Altered postural control was predominantly associated with increased co‐contraction of leg muscles. Body sway and leg and neck muscle co‐contraction correlated with the severity of subjective anxiety (*P* < 0.050). Alterations in postural control diminished if there were nearby stationary contrasts in the visual surrounding or if subjects stood with eyes closed. The performance of a cognitive dual task also improved impaired balance. Visual heights have two behavioral effects in vHI subjects: A change occurs in (1) open‐ and closed‐loop postural control strategy and (2) co‐contraction of anti‐gravity leg and neck muscles, both of which depend on the severity of evoked fear at heights.

## Abstract (precis)

We examine balance control and anti‐gravity muscle activity in persons susceptible to visual height intolerance (vHI) during the experience of fear at heights. Visual heights have two behavioral effects in vHI subjects: A change occurs in (1) open‐ and closed‐loop postural control strategy and (2) co‐contraction of anti‐gravity leg and neck muscles, both of which depend on the severity of evoked fear at heights.

## Introduction

Individual responses to visual stimulation of heights vary within a continuum ranging from physiological visual height imbalance to acrophobia, the severest end of the spectrum (Salassa and Zapala 2009). Physiological visual height imbalance is experienced by everyone and results from a mismatch between visual distance cues and the perception of self‐movement when the distance between the eyes and nearest objects in the environment reaches a certain threshold (Brandt et al. 1980; Salassa and Zapala 2009). Acrophobia is defined to be a specific phobia by ICD‐10 (WHO 1993) and DSM‐V (APA 2013), implying that an anticipatory fear leads to avoidance of heights. Acrophobia usually requires psychotherapy and has a lifetime prevalence of about 5% (LeBeau et al. 2010). In between the common physiological and the phobic reaction to heights, there is a stimulus‐dependent visual height intolerance (vHI), which causes the apprehension of losing balance or falling, but does not meet the diagnostic criteria of a specific phobia (Brandt et al. 2012a; Brandt and Huppert 2014). VHI is usually a life‐long, more or less distressing susceptibility to height stimuli, which affects 28% of the general population (Brandt et al. 2012b; Huppert et al. 2013). VHI can be very disabling and is known to have a relevant impact on the quality of life and daily activities of susceptible individuals (Schaeffler et al. 2013).

While the impact of physiological visual height imbalance (Bles et al. 1980; Adkin et al. 2002; Alpers and Adolph 2008) and acrophobia (Boffino et al. 2009; Hüweler et al. 2009) on posture has been investigated earlier, a quantitative assessment of postural alterations associated with vHI is still lacking. Persons susceptible to vHI experience subjective dizziness and postural to‐and‐fro vertigo when confronted with a height stimulus (Huppert et al. 2013). In patients suffering from another condition called somatoform phobic postural vertigo, it has been demonstrated that suchlike symptoms originate from an anxiety‐driven application of an inadequate postural control strategy (Wuehr et al. 2013). We hypothesized that an anxious, inadequately tuned control of posture may therefore also trigger subjective vertigo in subjects with vHI. Balance control requires the regulation of body sway to keep the body's line of gravity within certain balance boundaries. To detect alterations in the mechanisms governing balance control, a time‐series approach called stabilogram diffusion analysis (SDA) was introduced by Collins and De Luca (1993). SDA analysis of center‐of‐pressure (CoP) displacements indicates that open‐loop control governs postural behavior over short‐term intervals (< 2 s), whereas closed‐loop mechanisms regulate long‐term intervals (Collins and De Luca 1993, 1995; Collins et al. 1995). An open‐loop control system operates without sensory feedback and determines the steady‐state activity of anti‐gravity muscles (Laughton et al. 2003). Open‐loop feed‐forward control thus represents the motor commands that place the body in a desired posture. In contrast, closed‐loop control relies on sensory feedback from the visual, vestibular, and proprioceptive systems. Closed‐loop feedback control corrects drifts away from desired posture due to the effects of gravity, stochastic variations in muscle tone, etc. Intervention of feedback control might be triggered when CoP displacement exceeds certain boundaries (Collins and De Luca 1993) or when CoP velocity reaches a certain threshold (Delignieres et al. 2011). The time threshold where postural control switches from open‐ to closed‐loop behavior is delimited by the so‐called critical point.

The aim of this study was to explore alterations in postural control and muscle activity, which occur in subjects susceptible to vHI during height exposure and to determine if these alterations depend on visual stimulation and/or anxiety. We therefore evaluated balance control in vHI subjects while standing at heights (emergency balcony) versus standing at ground level (laboratory). Stimulus‐ and attention‐dependent changes in postural control were evaluated by a comprehensive stance protocol, including different visual feedback conditions and cognitive dual tasks. Postural behavior was analyzed by means of CoP amplitude variables (root mean square [RMS], sway range, and area) and SDA. Accompanying EMG was performed to evaluate associated changes in muscle activity levels and muscle cocontraction patterns. The major question was whether these subjects apply different strategies of postural control and patterns of anti‐gravity muscle innervation during free stance at ground level or when exposed to heights.

## Methods

### Subjects

Sixteen subjects (seven females; mean age 46.1 ± 15.0 years; mean height 174.4 ± 10.5 cm; mean weight 75.9 ± 18.3 kg) who had reported lifetime vHI participated in the study. A detailed questionnaire was used to enquire about vHI (Huppert et al. 2013). VHI subjects with acrophobia (to the extent of a specific phobia) or with past or current history of other psychiatric disorders (e.g., anxiety disorders, depression, or phobias) were excluded, as were participants with a known history of vestibular and balance deficits or other reported neurological or orthopedic disorders that affect postural control. All selected participants had normal or corrected‐to‐normal vision. All subjects gave their written informed consent prior to the experiments. The study protocol was approved by the local Ethics Committee and was conducted in conformity with the Declaration of Helsinki.

### Experimental procedure

**Figure 1.**
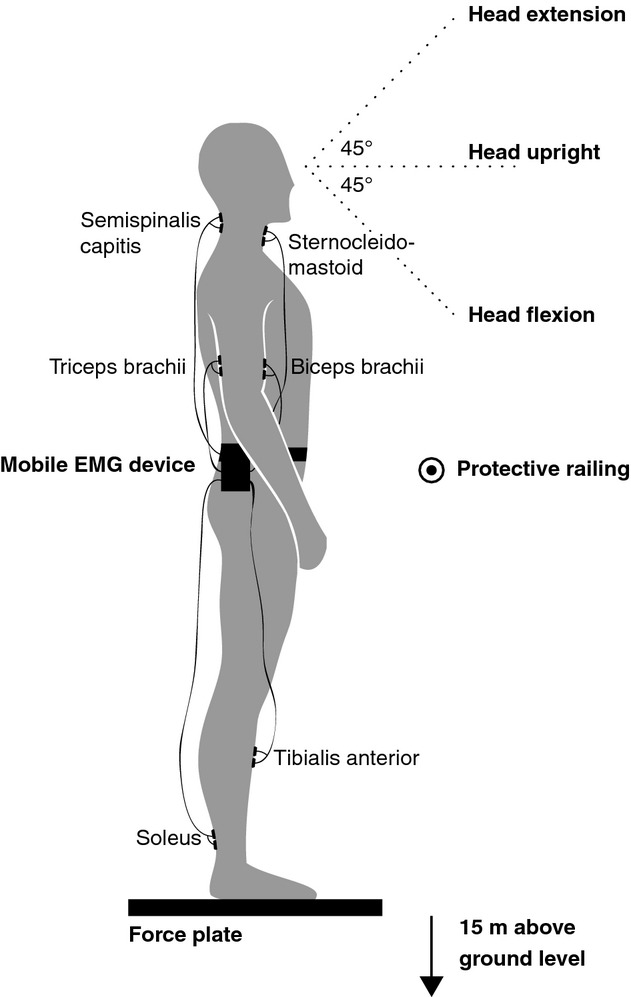
Experimental setup. Participants were asked to stand on a force plate on a 15‐m‐high balcony to assess center‐of‐pressure displacements. Electromyographic data of three muscle pairs were recorded with a mobile EMG device fixed around the waist: (1) tibialis anterior and soleus for the leg (2), biceps brachii and triceps brachii for the arm, and (3) sternocleidomastoid and semispinalis capitis for the neck. The stance protocol included the following conditions: (1) eyes open and normal head tilt, (2) eyes open and head flexion, (3) eyes open and head extension, (4) eyes closed, (5) eyes open while performing a cognitive dual task, and (6) eyes closed while performing a cognitive dual task. The complete stance protocol was repeated in the laboratory at ground level.

The postural performance of each participant was evaluated in two relevant situations: First while standing on an emergency balcony (height: 15 m) and afterward while standing in the laboratory. The experimental protocol for both measurements comprised six postural tasks during which each participant was instructed to stand quietly for 30 s: (1) Eyes open, line of gaze toward the horizon (EO); (2) eyes open, line of gaze toward a given object at ground level, that is, ↓ 45° head flexion in the pitch plane (EOHF); (3) eyes open, line of gaze toward the sky, that is, ↑ 45° head extension in the pitch plane (EOHE); (4) eyes closed (EC); (5) eyes open while performing a cognitive dual task (naming items from a given category) (EODT); and (6) eyes closed while performing a cognitive dual task (naming items from a given category) (ECDT). At the end of the protocol the first task (i.e., EO) was repeated to evaluate potential training and compensatory effects (Fig. 1).

After being measured on the balcony, each participant completed a standardized questionnaire. This included a subjective rating of the fear felt during height exposure (scale from 0 [no fear] to 10 [most severe form of fear]) and a report of any accompanying somatic symptoms and compensatory behavior. Furthermore, the impact of vHI on daily routine and quality of life was evaluated.

### Data recording

#### Posturography recordings

Static postural behavior was measured on a stabilometer platform (Type 9261A; Kistler, Winterthur, Switzerland) with a sampling frequency of 100 Hz. The CoP trajectories in anterior–posterior (AP) and medial–lateral (ML) directions were calculated and filtered using a second‐order low‐pass Butterworth filter with a cutoff frequency of 10 Hz to eliminate low‐amplitude measurement noise (Donker et al. 2007).

#### EMG recordings

Muscle activity was measured using surface EMG (Telemyo 2400, Noraxon USA Inc. Scottsdale, AZ) at a sampling frequency of 1500 Hz. To evaluate activity levels and co‐contraction of the leg, arm, and neck musculature, the following three muscle pairs were selected: (1) tibialis anterior and soleus for the leg (Nagai et al. 2012), (2) biceps brachii and triceps brachii for the arm (Song et al. 2013), and (3) sternocleidomastoid (SCM) and semispinalis capitis (SSC) for the neck (Cheng et al. 2008). Bipolar surface electrodes were placed on each of the selected muscles on the dominant leg side (Fig. 1). The ground electrode was affixed to the skin of the fibula head of the dominant leg. The raw EMG signal was band‐pass filtered at 20–500 Hz. The RMS amplitude of the signal was computed using a 50‐ms window. EMG activity of each examined muscle was additionally recorded during maximal voluntary contraction (MVC) (Kendall et al. 1993).

### Data analysis

#### Classic CoP parameters

RMS (mm) and range (mm) were calculated for AP and ML directions. The sway area (mm^2^) was estimated by fitting an ellipse covering 95% of the planar CoP displacement (Duarte and Zatsiorsky 2002).

#### Stabilogram diffusion analysis

**Figure 2.**
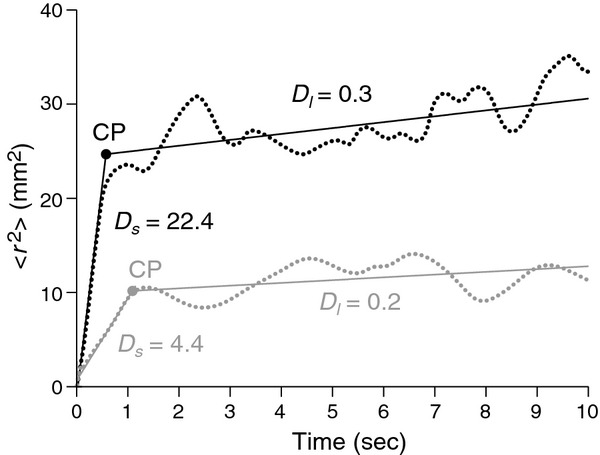
Sample stabilogram diffusion plot. Representative stabilogram diffusion plots (dotted lines) and fitted regressions (solid lines) of CoP displacement in the anterior–posterior direction of one individual susceptible to visual height intolerance, while quietly standing on the balcony (black lines) and while quietly standing in the laboratory at ground level (gray lines). The short‐term diffusion coefficient *D*_s_, the long‐term diffusion coefficient *D*_l_, and the critical point CP are shown for both stance conditions. During height exposure, the short‐term range shows an increased diffusion activity, indicating abnormal open‐loop control. In addition, the critical time interval CP, at which short‐term behavior changes into long‐term behavior, is shortened, indicating a precipitate intervening of closed‐loop control into the postural control scheme.

SDA analysis was performed for the CoP displacement in AP and ML directions. The CoP SDAs were calculated with the following equation:
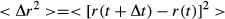
where <·> indicates the calculation of the mean of the time series. This computation is repeated for increasing values of Δ*t* in the range 0–10 s. The resulting diffusion plot shows the mean squared displacements against the time intervals Δ*t*. The short‐ and long‐term diffusion coefficients *D*_s_ (mm^2^s^−1^) and *D*_l_ (mm^2^s^−1^) were determined by linear fits to the diffusion plot. In most cases, the linear fit to the short‐term region included data ranging from 0.1 to 0.5 s; the linear fit to the long‐term region included data ranging from 2 to 5 s. In some cases fitting regions were manually altered to maintain good‐quality linear fits of the data. The critical point coordinates Δ*t*_c_ (s) (critical time) and <Δr^2^>_c_ (mm^2^) (critical displacement) were obtained from the intersection point of the linear fits to the short‐ and long‐term regions (Fig. 2). The scaling exponents for the short‐ and long‐term region *H*_s_ and *H*_l_ were determined by linear fits to the log–log plot of the SDA. These exponents, which lie in the range 0 < *H *<**1, quantify the correlation between the step increments, which make up the stabilogram time series. For *H *>**0.5, past and future increments are positively correlated. For *H *<**0.5, past and future increments are negatively correlated.

#### EMG analysis

MVC recordings were used to normalize the EMG amplitude of each muscle during the postural tasks. To evaluate the relative level of co‐contraction within the selected leg, arm, and neck muscle pairs, the co‐contraction index (CI) was calculated for each pair following the approach proposed by Falconer and Winter (Falconer and Winter 1985; Nagai et al. 2012):
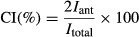
*I*_ant_ is the area of the total antagonistic activity calculated by:
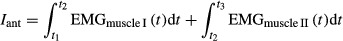
where *t*_1_ to *t*_2_ designates the period in which the muscle I EMG is less than the muscle II EMG and *t*_2_ to *t*_3_ designates the period in which the muscle II EMG is less than the muscle I EMG. *I*_total_ is the integral of the sum of muscle I and muscle II EMG over the whole measurement period:
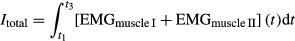


### Statistical analysis

The effects of each dependent variable were analyzed using a three‐way repeated measurement analysis of variance (ANOVA) and a Bonferroni post hoc analysis with height stimulus (balcony vs. laboratory), stance condition (EO, EOHF, EOHE, EC, EODT, ECDT), and CoP displacement direction (AP vs. ML direction) as factors. Significant interaction effects were further decomposed into simple main effects. Pearson's correlations were performed to evaluate (1) the influence of muscle activity and co‐contraction on the CoP variables and (2) the influence of subjectively rated anxiety on the EMG and posturography outcomes during height exposure. The resulting *P* values were corrected for multiple comparisons using the false discovery rate (FDR) procedure (Benjamini and Hochberg 1995). Results were considered significant, if *P *<**0.05. Statistical analysis was performed using SPSS (Version 20.0; IBM Corp., Armonk, NY).

## Results

ANOVA results are given in Table 1 and correlation analysis outcomes in Table 2. Descriptive statistics (mean ± SD) are presented in Tables 3 and 4.

**Table 1.** Results of the two‐way ANOVA for the EMG and body sway parameters.

	Height (balcony/lab)	Condition	Direction (AP/ML)	Height × condition
*Classic CoP parameters*				
Range	***F***_**1,28**_ ** = 13.2, ** ***P *** **=** ****** **0.001**	***F***_**6,168**_ ** = 3.8, ** ***P *** **=** ****** **0.013**	***F***_**1,28**_ ** = 24.7, ** ***P < *** **0.001**	*F*_6,168_ = 2.4, *P = *0.530
RMS	*F*_1,28_ = 1.0, *P *=**0.322	***F***_**6,168**_ ** = 8.9, ** ***P *** **<** ****** **0.001**	***F***_**1,28**_ ** = 14.1, ** ***P = *** **0.001**	*F*_6,168_ = 1.9, *P = *0.128
Area	*F*_1,14_ = 0.1, *P *=**0.747	*F*_6,84_ = 3.4, *P *=**0.067		*F*_6,84_ = 1.6, *P = *0.156
*Stabilogram diffusion analysis parameters*				
Diffusion coefficients				
*D*_s_	***F***_**1,28**_ ** = 26.8, ** ***P *** **<** ****** **0.001**	***F***_**6,168**_ ** = 3.4, ** ***P *** **=** ****** **0.019**	***F***_**1,28**_ ** = 5.6, ** ***P = *** **0.025**	***F***_**6,168**_ ** = 5.3, ** ***P = *** **0.002**
*D*_l_	*F*_1,28_ = 1.1, *P *=**0.311	*F*_6,168_ = 1.5, *P *=**0.206	***F***_**1,28**_ ** = 6.7, ** ***P = *** **0.015**	*F*_6,168_ = 1.3, *P *=**0.263
Scaling exponents				
*H*_s_	*F*_1,28_ = 0.8, *P *=**0.373	*F*_6,168_ = 0.4, *P *=**0.891	*F*_1,28_ = 0.1, *P = *0.987	*F*_6,168_ = 1.2, *P = *0.294
*H*_l_	***F***_**1,28**_ ** = 5.8, ** ***P *** **=** ****** **0.023**	*F*_6,168_ = 3.3, *P *=**0.098	*F*_1,28_ = 0.1, *P = *0.832	*F*_6,168_ = 0.7 *P = *0.619
Critical point coordinates				
*∆t*_c_	***F***_**1,28**_ ** = 12.5, ** ***P *** **=** ****** **0.001**	*F*_6,168_ = 1.6, *P *=**0.137	*F*_1,28_ = 1.3, *P = *0.256	***F***_**6,168**_ ** = 2.7, ** ***P *** **=** ****** **0.030**
<*∆r*^2^>_c_	*F*_1,28_ = 0.1, *P *=**0.786	***F***_**6,168**_ ** = 8.6, ** ***P *** **=** ****** **0.001**	***F***_**1,28**_ ** = 8.6, ** ***P = *** **0.007**	*F*_6,168_ = 0.5, *P *=**0.576
*EMG parameters*				
Normalized EMG activity				
Soleus	*F*_1,14_ = 3.7, *P *=**0.076	*F*_6,84_ = 1.7, *P *=**0.168		*F*_6,84_ = 3.5, *P *=**0.741
Tib. Ant.	***F***_**1,14**_ ** = 9.0, ** ***P *** **=** ****** **0.010**	***F***_**6,84**_ ** = 3.6, ** ***P *** **=** ****** **0.001**		***F***_**6,84**_ ** = 3.9, ** ***P *** **<** ****** **0.001**
Biceps	*F*_1,14_ = 1.4, *P *=**0.255	*F*_6,84_ = 2.2, *P *=**0.132		*F*_6,84_ = 2.1, *P *=**0.155
Triceps	*F*_1,14_ = 0.2, *P *=**0.704	*F*_6,84_ = 0.5, *P *=**0.650		*F*_6,84_ = 3.4, *P *=**0.092
SCM	*F*_1,14_ = 1.0, *P *=**0.328	*F*_6,84_ = 2.5, *P *=**0.098		*F*_6,84_ = 0.5, *P *=**0.670
SSC	*F*_1,14_ = 0.1, *P *=**0.930	*F*_6,84_ = 0.9, *P *=**0.420		*F*_6,84_ = 0.5, *P *=**0.553
Co‐contraction index				
Leg CI	*F*_1,14_ ** = 10.6, ** ***P *** **=** ****** **0.006**	***F***_**6,84**_ ** = 4.2, ** ***P *** **=** ****** **0.009**		***F***_**6,84**_ ** = 5.1, ** ***P *** **=** ****** **0.002**
Arm CI	*F*_1,14_ = 4.5, *P *=**0.051	*F*_6,84_ = 2.1, *P *=**0.119		*F*_6,84_ = 0.9, *P *=**0.859
Neck CI	*F*_1,14_ = 0.1, *P *=**0.983	*F*_6,84_ = 1.3, *P *=**0.274		*F*_6,84_ = 0.8, *P *=**0.536

Significant height, condition, direction, and height × condition effects are marked in bold. AP, anterior–posterior; ML, medial–lateral; RMS, root mean square; *D*_s_, short‐term diffusion coefficient; *D*_l_, long‐term diffusion coefficient; *H*_s_, short‐term scaling exponent; *H*_l_, long‐term scaling exponent; *∆t*_c_, critical time; <*∆r*^2^>_c_, critical displacement; SCM, sternocleidomastoid muscle; SSC, semispinalis capitis muscle; CI, co‐contraction index.

**Table 2.** Correlations between EMG measures and postural control parameters (A) and correlations between the subjective estimates of fear at heights and the EMG as well as the postural control measures while standing on the balcony (B).

(A)	Soleus	Tib. Ant.	Biceps	Triceps	SCM	SSC	Leg CI	Arm CI	Neck CI
Classic CoP parameters									
Range	−0.057	**0.149** *	0.114	0.043	−0.051	0.067	**0.216** *	−0.008	0.021
RMS	0.115	0.020	0.127	0.106	−0.001	0.036	0.090	0.019	−0.018
Area	0.102	0.032	0.143	0.124	−0.045	−0.055	0.106	0.016	−0.020
Stabilogram diffusion analysis parameters									
*D*_s_	0.014	**0.161** *	0.109	0.119	−0.099	−0.002	**0.285** *	−0.058	−0.087
*D*_l_	**0.149** *	0.012	0.031	0.150	0.053	0.097	0.044	0.054	−0.100
*H*_s_	0.098	−0.002	−0.047	0.068	−0.004	0.087	0.001	0.044	0.011
*H*_l_	0.041	−0.075	−0.053	−0.075	0.068	0.042	−0.072	0.067	−0.006
*∆t*_c_	−0.017	−0.080	−0.167*	−0.096	0.065	−0.088	−0.063	0.101	0.023
<*∆r*^2^>_c_	0.124	−0.021	0.143	0.062	0.034	0.009	−0.002	0.039	−0.006

(B) EMG parameters								
Soleus	Tib. Ant.	Biceps	Triceps	SCM	SSC	Leg CI	Arm CI	Neck CI
−0.343*	**0.371** *	**0.190** *	−0.176*	0.007	−0.081	**0.281** *	−0.108	**0.141** *
Classic CoP and stabilogram diffusion analysis parameters								
Range	RMS	Area	*D*_s_	*D*_l_	*H*_s_	*H*_l_	*∆t*_c_	<*∆r*^2^>_c_
**0.156** *	−0.070	0.018	**0.164** *	−0.063	−0.031	−0.067	−0.069	−0.138

Pearson's correlation coefficient (r). Significant correlations are marked in bold. SCM, sternocleidomastoid muscle; SSC, semispinalis capitis muscle; CI, co‐contraction index; RMS, root mean square; *D*_s_, short‐term diffusion coefficient; *D*_l_, long‐term diffusion coefficient; *H*_s_, short‐term scaling exponent; *H*_l_, long‐term scaling exponent; *∆t*_c_, critical time; <*∆r*^2^>_c_, critical displacement.

* A significant correlation *P *<**0.05.

**Table 3.** Descriptive statistics (mean ± SD) of the classic CoP and stabilogram diffusion analysis parameters.

Classic CoP parameters					
	Range (mm)		RMS (mm)		Area (mm^2^)
	ML	AP	ML	AP	
Balcony					
EO	34.9 ± 30.1	51.9 ± 26.4	3.7 ± 1.6	5.3 ± 2.4	205.3 ± 138.5
EOHF	35.0 ± 23.7	43.4 ± 33.3	3.3 ± 1.1	3.9 ± 1.5	150.6 ± 94.5
EOHE	31.7 ± 15.1	53.9 ± 22.5	4.5 ± 2.0	7.2 ± 3.1	403.4 ± 317.8
EC	29.2 ± 13.3	55.8 ± 20.4	3.8 ± 0.9	6.5 ± 2.8	307.1 ± 220.4
EODT	35.9 ± 15.7	66.2 ± 31.5	5.0 ± 2.4	6.4 ± 5.2	391.8 ± 268.2
ECDT	38.8 ± 25.8	66.6 ± 28.3	6.1 ± 4.9	8.0 ± 1.8	340.7 ± 212.5
Laboratory					
EO	21.1 ± 6.8	38.7 ± 18.9	3.3 ± 1.1	5.0 ± 2.5	201.8 ± 159.0
EOHF	20.3 ± 10.4	39.2 ± 18.8	3.3 ± 2.0	5.4 ± 3.1	225.0 ± 236.2
EOHE	21.1 ± 8.2	36.6 ± 18.1	3.3 ± 1.9	4.9 ± 2.3	215.6 ± 208.4
EC	22.1 ± 7.6	43.9 ± 18.0	3.4 ± 1.7	6.3 ± 3.6	311.6 ± 295.0
EODT	31.4 ± 23.0	39.1 ± 17.1	4.5 ± 2.6	6.0 ± 3.6	390.1 ± 497.9
ECDT	35.1 ± 22.5	44.0 ± 20.1	6.0 ± 4.3	7.5 ± 4.3	374.8 ± 277.7

Stabilogram diffusion analysis parameters						
	*D*_s_ (mm^2^s^−1^)		*D*_l_ (mm^2^s^−1^)		*H*_s_	
	ML	AP	ML	AP	ML	AP
Balcony						
EO	29.8 ± 26.8	29.3 ± 19.6	1.1 ± 1.2	0.8 ± 0.8	0.83 ± 0.15	0.88 ± 0.08
EOHF	8.4 ± 9.7	11.6 ± 13.3	0.7 ± 0.7	0.9 ± 0.6	0.86 ± 0.07	0.89 ± 0.05
EOHE	18.7 ± 16.1	28.0 ± 19.7	1.2 ± 2.3	2.0 ± 1.5	0.88 ± 0.12	0.84 ± 0.15
EC	15.2 ± 12.8	26.3 ± 16.5	1.2 ± 1.1	1.6 ± 1.4	0.89 ± 0.08	0.86 ± 0.08
EODT	9.8 ± 7.4	25.4 ± 27.3	0.6 ± 1.1	1.7 ± 2.4	0.84 ± 0.12	0.85 ± 0.15
ECDT	18.2 ± 16.6	25.1 ± 14.5	1.6 ± 1.9	1.6 ± 1.9	0.85 ± 0.09	0.90 ± 0.04
Laboratory						
EO	4.9 ± 3.0	6.6 ± 2.8	1.0 ± 1.3	2.0 ± 1.9	0.85 ± 0.10	0.86 ± 0.12
EOHF	6.5 ± 6.2	11.8 ± 9.7	1.1 ± 1.2	1.6 ± 2.6	0.89 ± 0.06	0.84 ± 0.15
EOHE	7.5 ± 6.4	11.2 ± 9.5	0.8 ± 1.1	1.5 ± 1.9	0.86 ± 0.10	0.89 ± 0.08
EC	15.0 ± 14.2	16.6 ± 11.5	0.6 ± 0.5	1.4 ± 1.6	0.88 ± 0.08	0.87 ± 0.05
EODT	8.9 ± 6.7	21.6 ± 46.6	1.3 ± 1.3	2.5 ± 3.9	0.89 ± 0.05	0.86 ± 0.11
ECDT	20.2 ± 18.1	24.7 ± 20.0	1.6 ± 1.9	2.3 ± 2.4	0.90 ± 0.05	0.87 ± 0.09
	*H*_l_		*∆t*_c_ (s)		<*∆r*^2^>_c_ (mm^2^)	
	ML	AP	ML	AP	ML	AP
Balcony						
EO	0.14 ± 0.07	0.16 ± 0.14	0.6 ± 0.4	0.9 ± 0.4	14.4 ± 11.3	44.7 ± 54.1
EOHF	0.20 ± 0.12	0.19 ± 0.14	1.3 ± 1.5	1.3 ± 1.2	14.1 ± 14.1	25.7 ± 32.6
EOHE	0.16 ± 0.11	0.15 ± 0.14	1.1 ± 0.7	1.3 ± 0.8	29.9 ± 27.1	85.5 ± 74.0
EC	0.15 ± 0.16	0.14 ± 0.10	1.2 ± 1.3	1.6 ± 0.7	25.0 ± 15.0	74.9 ± 53.5
EODT	0.12 ± 0.09	0.12 ± 0.07	1.2 ± 0.8	1.2 ± 0.4	42.0 ± 48.3	64.0 ± 32.0
ECDT	0.14 ± 0.10	0.13 ± 0.08	1.3 ± 1.3	1.1 ± 0.6	113.5 ± 214.9	143.8 ± 230.7
Laboratory						
EO	0.16 ± 0.11	0.20 ± 0.12	1.9 ± 1.0	1.4 ± 0.8	21.9 ± 19.9	40.3 ± 55.7
EOHF	0.21 ± 0.10	0.19 ± 0.11	1.2 ± 0.8	1.8 ± 1.1	15.5 ± 15.5	42.8 ± 57.8
EOHE	0.21 ± 0.12	0.23 ± 0.14	1.6 ± 1.2	2.1 ± 1.3	17.8 ± 14.1	47.0 ± 50.8
EC	0.14 ± 0.12	0.18 ± 0.12	1.2 ± 1.1	1.8 ± 1.2	28.4 ± 33.8	96.8 ± 183.7
EODT	0.15 ± 0.09	0.17 ± 0.12	1.1 ± 0.8	1.2 ± 0.9	41.6 ± 51.9	55.2 ± 54.7
ECDT	0.17 ± 0.09	0.14 ± 0.10	1.2 ± 0.8	1.5 ± 1.2	61.7 ± 61.8	144.5 ± 196.4

AP, anterior–posterior, ML, medial–lateral; RMS, root mean square; *D*_s_, short‐term diffusion coefficient; *D*_l_, long‐term diffusion coefficient; *H*_s_, short‐term scaling exponent; *H*_l_, long‐term scaling exponent; *∆t*_c_, critical time; <*∆r*^2^>_c_, critical displacement; EO, eyes open and normal head tilt; EOHF, eyes open and head flexion; EOHE, eyes open and head extension; EC, eyes closed; EODT, eyes open while performing a cognitive dual task; ECDT, eyes closed while performing a cognitive dual task.

**Table 4.** Descriptive statistics (mean ± SD) of the EMG parameters.

	Soleus (%)	Tib. Ant. (%)	Biceps (%)	Triceps (%)	SCM (%)	SSC (%)	Leg CI (%)	Arm CI (%)	Neck CI (%)
Balcony									
EO	14.2 ± 17.2	2.7 ± 2.4	1.5 ± 1.3	1.5 ± 1.1	3.5 ± 3.3	5.9 ± 3.6	30.9 ± 24.2	52.9 ± 27.7	39.7 ± 21.0
EOHF	14.2 ± 17.2	2.3 ± 3.3	1.1 ± 1.0	1.4 ± 1.1	3.7 ± 3.5	6.9 ± 5.8	13.7 ± 8.9	58.2 ± 26.3	38.0 ± 21.4
EOHE	14.7 ± 16.2	4.6 ± 5.9	1.0 ± 0.8	1.5 ± 1.3	3.4 ± 1.6	6.2 ± 4.7	30.2 ± 21.6	56.0 ± 26.0	36.0 ± 22.1
EC	14.3 ± 15.3	4.7 ± 4.3	1.0 ± 0.8	1.7 ± 1.3	2.7 ± 1.7	5.9 ± 5.6	33.2 ± 23.8	60.6 ± 27.4	40.9 ± 22.8
EODT	15.7 ± 16.6	2.1 ± 3.2	1.1 ± 1.3	1.1 ± 1.0	5.3 ± 5.3	7.3 ± 6.4	16.5 ± 10.5	51.3 ± 29.6	43.1 ± 22.4
ECDT	18.0 ± 18.3	2.7 ± 5.3	1.0 ± 0.9	1.0 ± 0.8	4.8 ± 4.2	7.7 ± 7.5	19.9 ± 15.7	56.6 ± 26.6	42.9 ± 26.5
Laboratory									
EO	22.7 ± 26.5	0.9 ± 0.6	0.9 ± 0.8	1.0 ± 0.8	2.3 ± 1.1	5.9 ± 4.9	10.4 ± 7.1	64.7 ± 22.2	40.7 ± 24.5
EOHF	21.8 ± 24.7	0.9 ± 0.6	0.9 ± 0.9	1.1 ± 0.9	5.0 ± 7.6	8.6 ± 10.1	12.5 ± 13.1	68.5 ± 20.1	36.4 ± 24.8
EOHE	22.7 ± 27.1	1.3 ± 1.6	0.9 ± 0.9	1.1 ± 0.8	4.9 ± 5.7	7.4 ± 10.4	10.5 ± 6.6	66.0 ± 19.3	39.3 ± 28.9
EC	22.3 ± 25.1	1.2 ± 0.8	0.9 ± 0.9	1.0 ± 0.8	3.3 ± 2.9	4.8 ± 6.2	12.9 ± 8.1	66.5 ± 22.8	39.4 ± 24.7
EODT	22.7 ± 27.6	2.1 ± 4.8	1.0 ± 0.9	1.7 ± 2.1	7.2 ± 7.5	6.7 ± 5.6	10.5 ± 7.9	56.1 ± 24.4	47.6 ± 22.2
ECDT	24.4 ± 27.2	1.1 ± 0.8	1.0 ± 0.8	1.3 ± 1.1	4.6 ± 4.2	7.2 ± 7.1	11.0 ± 8.7	60.1 ± 25.2	45.7 ± 23.7

SCM, sternocleidomastoid muscle; SSC, semispinalis capitis muscle; CI, co‐contraction index; EO, eyes open and normal head tilt; EOHF, eyes open and head flexion; EOHE, eyes open and head extension; EC, eyes closed; EODT, eyes open while performing a cognitive dual task; ECDT, eyes closed while performing a cognitive dual task.

### Questionnaire outcomes

Subjectively rated anxiety while standing on the balcony averaged 4.6 ± 2.5 (min = 0; max = 10). Among the accompanying somatic symptoms, instability of stance/gait was most frequently reported (64%) followed by inner agitation (57%), to‐and‐fro vertigo and trembling (both 43%), queasy‐stomach feeling (36%), weakness in the knees (29%), fearfulness and sudden sweating (both 21%), palpitations and light headedness (14%). The most common compensatory behavior was thinking about a safe grip (57%), avoiding looking down (43%), staying close to the building walls (29%), and considering withdrawing from the experiment (21%). A total of 36% of the participants indicated that aside from typical triggering situations, vHI had no disturbing impact on their daily routine. On the other hand, 64% reported that in typical triggering situations vHI led to compensatory behavior with relevant consequences for their quality of life.

### Body sway parameters

None of the examined CoP parameters showed any significant training effects for the duration of the experiments. Sway ranges were greater during height exposure. Under the condition EOHF, ranges were generally smaller than for the condition ECDT. In contrast, CoP RMS and sway area did not differ significantly between the different height situations. RMS was generally smaller while standing under the condition EOHF compared to the conditions EOHE and ECDT. Both sway ranges and RMSs were higher in AP than in the ML direction.

**Figure 3.**
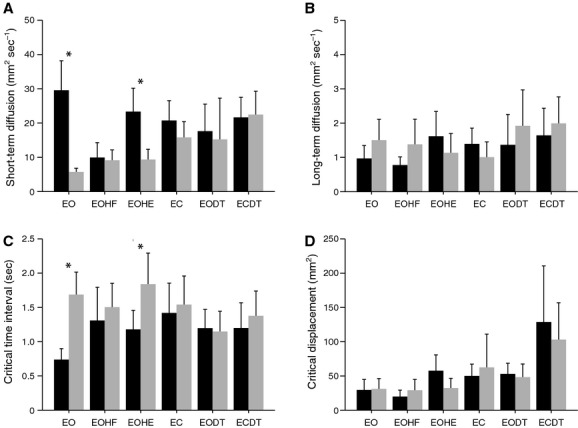
Stabilogram diffusion analysis parameters. (A) the short‐term (open‐loop) diffusion coefficient *D*_s_, (B) the long‐term (closed‐loop) diffusion coefficient *D*_l_, (C) the critical time interval *∆t*_c_, and (D) the critical mean squared displacement <*∆r*^2^>_c_. Standing on the balcony (black bars), standing in the laboratory (gray bars). Stance conditions: eyes open and normal head tilt (EO), eyes open and head flexion (EOHF), eyes open and head extension (EOHE), eyes closed (EC), eyes open while performing a cognitive dual task (EODT), and eyes closed while performing a cognitive dual task (ECDT). During height exposure under the conditions EO and EOHE postural control is altered in (1) increased short‐term diffusion activity, indicating abnormal open‐loop control, and (2) shortened critical time intervals, indicating that the primary sensory feedback threshold of the postural control system is lowered.

SDA yielded higher *D*_s_ during height exposure. Decomposing the significant interaction effect revealed that increased *D*_s_ was only present for the conditions EO and EOHE, but comparable for all other stance conditions. While standing on the balcony, *D*_s_ was decreased under the condition EOHF compared to all other conditions except EODT. In contrast, *D*_l_ results were in the same range for both height situations and all stance conditions. Overall, *H*_s_ was similar for the different height stimuli and stance conditions. *H*_l_ was smaller during height exposure, but did not differ between stance conditions. Critical point analysis revealed smaller Δ*t*_c_ during height exposure. Decomposing the significant interaction effect revealed that shortened Δ*t*_c_ was only present under the conditions EO and EOHE, but comparable for all other stance conditions. <Δr^2^>_c_ was comparable between both height situations, but was generally decreased under the condition EOHF compared to the conditions EODT and ECDT. *D*_s_, *D*_l_, as well as <Δr^2^>_c_ were generally higher in AP than in the ML direction (Fig. 3).

### EMG measures

None of the analyzed EMG measures showed any significant training effects for the duration of the experiments. Mean normalized EMG activity of the soleus, biceps brachii, triceps brachii, SCM, and SSC did not show any significant changes between different height stimuli and all examined stance conditions. In contrast, mean normalized muscle activity of the tibialis anterior was markedly increased during height exposure. Decomposing the significant interaction effect revealed that increased tibialis anterior activity was only present for the conditions EO, EOHE, and EC. While subjects stood on the balcony, tibialis anterior activity was higher under the condition EC compared to the conditions EO, EOHF, and EODT.

**Figure 4.**
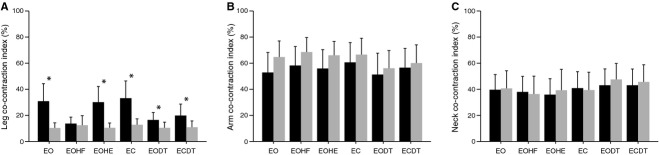
Co‐contraction muscle activity. Co‐contraction index of (A) the leg muscles (i.e., tibialis anterior and soleus muscle), (B) the arm muscles (i.e., biceps brachii and triceps brachii muscle), and (C) the neck muscles (i.e., sternocleidomastoid and semispinalis capitis muscle). Stance conditions: eyes open and normal head tilt (EO), eyes open and head flexion (EOHF), eyes open and head extension (EOHE), eyes closed (EC), eyes open while performing a cognitive dual task (EODT), and eyes closed while performing a cognitive dual task (ECDT). During height exposure co‐contraction increases in leg muscles under all stance conditions except EOHF.

Leg muscle co‐contraction was increased during height exposure. Decomposing the significant interaction effect revealed that increased leg muscle CI was present for all stance conditions except EOHF. On the balcony, leg muscle CI was higher under the conditions EO, EOHE, and EC compared to the conditions EOHF, EODT, and ECDT. In contrast, arm and neck muscle co‐contraction was similar for the different height stimuli and stance conditions (Fig. 4).

### Correlation analysis

Evaluating Pearson's correlations between EMG and posturography measures revealed moderate but significant correlations, predominantly between leg muscle activity and CoP parameters. Increased tibialis anterior activity and leg CI were associated with increased sway ranges and higher *D*_s_. In contrast, soleus activity positively correlated with *D*_l_. Furthermore, arm muscle activity of the biceps brachii positively correlated with Δ*t*_c_ (Table 2A).

Correlations between the subjective estimates of fear at heights, and the CoP and EMG measures during height exposure were also evaluated. The strongest positive correlations were found in the EMG measures of tibialis anterior activity and leg CI and in the CoP measures of range and *D*_s_, followed by moderate positive associations with biceps brachii activity and neck CI. Negative correlations were found for soleus and triceps brachii activity (Table 2B).

## Discussion

An analysis of body sway and muscle activity in subjects with vHI revealed a characteristic steady‐state behavior and the functional interactions of neuromuscular mechanisms underlying postural control in vHI. At heights, these subjects exhibited inadequate postural control, that is, the threshold for closed‐loop sensory feedback was lowered and open‐loop control was increased. These alterations were associated with specific changes in muscle activity parameters. The underlying mechanisms and the functional relevance of these findings for balance during quiet stance will be discussed in two parts: (1) The alterations in postural control measures and (2) the associated changes in muscle activity parameters.

### Postural control measures

Sway ranges increased in vHI subjects while quietly standing at heights in accordance with previous studies on healthy subjects (Alpers and Adolph 2008). Thus, height is a threat linked to increased oscillatory behavior, indicating an altered control scheme, which tends to shift the body's gravity line closer to the balance boundaries, making it more difficult to counteract external perturbations (Duarte and Zatsiorsky 2002). The SDA framework allows us to delve further into the regulatory modes underlying altered balance behavior in vHI subjects at heights. In accordance with previous studies on SDA (Collins and De Luca 1993; Collins et al. 1995), diffusion coefficients and scaling exponents generally revealed two distinct neuromuscular control mechanisms – one that acts over short‐term intervals and is governed by open‐loop control and one that acts over long‐term intervals and utilizes closed‐loop control. During height exposure, vHI subjects exhibited significant alterations in the steady‐state behavior of open‐loop control, while closed‐loop activity remained normal. The observed increase in *D*_s_ has been associated with an increase in postural instability, that is, a less tightly regulated and less damped postural control system (Collins and De Luca 1993; Collins et al. 1995; Peterka 2000). In addition, the location of the critical point, that is, the transition from open‐ to closed‐loop control, was shifted to smaller time intervals. The coordinates of the critical point have been associated with the first‐level stability limit of the postural control system, that is, its primary feedback threshold (Collins and De Luca 1993). The decrease in Δ*t*_c_ during height threat significantly shortens the effective range of the steady‐state open‐loop regime and thereby lowers the temporal primary feedback threshold. A decreased critical time has also been linked to an increased stiffness of the postural control system (Peterka 2000). During normal balance control, the complexity of the stochastic short‐term steady‐state behavior allows the postural control system to adapt closed‐loop responses to sudden balancing stresses and thus enhances postural stability. The limitations of the open‐loop mechanisms are linked to a prevalence of closed‐loop processes within postural control and have been associated with a functional decline in the system (Lipsitz 2002). Limited open‐loop control in vHI subjects triggers precipitate integration of sensory feedback into the postural control scheme and may lead to maladaptive responses to perturbations.

Almost all observed significant alterations of balance behavior were associated with the subjective estimates of fear at heights. This suggests that the degree of manifestation of altered postural control and the amount of anxiety experienced during height threat are mutually linked in vHI subjects, leading to a vicious circle of fear, perception, and instability (Schaeffler et al. 2013). Moreover, it was reported that fear at heights in persons susceptible to vHI also leads to restricted visual exploration, suggesting an anxiety‐driven visual avoidance behavior (Kugler et al. 2013). Altered postural control during height exposure was only present when looking toward the horizon or the sky (EO and EOHE) when the distance between the stationary contrast in the surround and the eyes was critically large. While optimal balance during free stance requires the continuous evaluation of the reafferent sensory feedback of self‐generated body movements, critically increased eye–object distance results in a mismatch between visual distance cues and the perception of self‐movement, thus causing postural imbalance (Bles et al. 1980; Brandt et al. 1980). Accordingly, alterations in postural control mainly disappeared either while looking at the ground (EOHF) when nearby stationary cues in the periphery of the visual field were provided (i.e., protective railing, objects on the ground level) or while standing with EC. These observations support the supposition that physiological visual height imbalance triggers postural disequilibrium in vHI. However, impaired balance control during height threat also diminished while performing a cognitive dual task (EODT and ECDT) when the vHI subject was distracted from the height threat. Increased attention to postural control has been associated with an anxious control of posture (Maki and McIlroy 1996). This observation indicates that visual environment and anxiety have a twofold effect, resulting in inadequate control of posture in vHI (Tersteeg et al. 2012).

### Muscle activity parameters

Altered postural control during height exposure was linked to specific changes in muscle activity parameters. Increased leg muscle activity predominantly accompanied alterations in the steady‐state open‐loop control and positively correlated with subjective estimates of fear at heights. Raised leg muscle co‐contraction observed in vHI subjects was mediated by a parallel downgrade of soleus activity and upgrade of tibialis anterior activity and in conformance with previously reported changes in leg muscle activity in healthy subjects standing under height threat (Brown and Frank 1997; Carpenter et al. 2001). This observation is in line with the supposition that increased stiffness of the musculoskeletal system due to increased muscular activity across the joints of the lower limbs could account for increased levels of short‐term stochastic activity (Collins and De Luca 1995; Laughton et al. 2003). It is well known that the force output of skeletal muscles contains noise‐like fluctuations (De Luca et al. 1982), which increase with muscle activity (Galganski et al. 1993). Larger noise‐like fluctuations over joints due to increased levels of muscle activity would therefore lead to increased short‐term postural sway and impaired postural control (Gruneberg et al. 2004; de Freitas et al. 2009). The observed changes in leg muscle activation during height exposure might be the result of leaning further away from the balcony edge (Carpenter et al. 2001; Pasman et al. 2011). In contrast, arm muscle activity did not show increased co‐contraction during height threat. Instead, increased biceps brachii and decreased triceps brachii activity positively correlated with subjective estimates of fear at heights, indicating that vHI subjects exhibit increased elbow joint flexion during height threat. This observation supposes that anticipatory arm movements occur, which might facilitate restabilization during impaired balance control (Maki and McIlroy 1997; Allum et al. 2002). This includes potential grasping movements toward the protective railing. Alterations in arm muscle activity were predominantly associated with the second characteristic change in postural control strategy, that is, the decreased temporal feedback threshold. Finally, the subjective estimates of fear at heights and co‐contraction of neck muscles were moderately, positively correlated. This finding agrees with the recent report that spontaneous head movements in vHI subjects are reduced during height exposure (Kugler et al. 2013). An increased stiffness and immobilization of the neck is thought to reduce postural stability (Koskimies et al. 1997). It has been argued that surface EMG may face difficulties in isolating pure SSC activity and is more likely to record the activity of a more general neck extensor group (including SSC and trapezius) (Joines et al. 2006). However, this would not undermine the conclusion that an increased relative co‐contraction of the SCM and the more general head extensor muscle group results in a stiffening of the neck. In summary, persons susceptible to vHI during height threat exhibit enhanced relative co‐contraction of anti‐gravity leg and neck muscles, which impacts on their steady‐state open‐loop control of posture. In contrast, increased bending of the arms may trigger precipitate sensory feedback control of posture and may serve to restabilize impaired balance.

This study provides first insights into the characteristic changes in postural control strategy in persons susceptible to vHI during height exposure. These changes appear to be predominantly linked to enhanced co‐contraction of anti‐gravity muscles. Two distinct sources of postural imbalance were identified: Both a critical distance to stationary surroundings in the visual environment and the anxiety evoked by height threat appear to trigger inadequate balance strategy in vHI subjects. The specificity of the observed alterations in balance control of these subjects shows a notable conformance with recently found changes in postural behavior in patients with phobic postural vertigo (Wuehr et al. 2013). This suggests that a general anxiety‐driven rather than a height‐specific motor reaction may underlie these two distinct forms of subjective vertigo.

## Acknowledgments

The authors thank Judy Benson for copyediting the manuscript.

## Conflict of Interest

None declared.
